# Terlipressin-induced hyponatraemia

**DOI:** 10.1186/cc13349

**Published:** 2014-03-17

**Authors:** R Gray, J Kilic, P Anderson

**Affiliations:** 1 BSUH, Brighton, UK

## Introduction

Terlipressin is a vasopressin (V1 receptor) agonist that causes splanchnic constriction and is used in the management of variceal bleeding. This case report demonstrates the profound hyponatraemia, sufficient to cause a fall in conscious level, developing following terlipressin administration for variceal gastrointestinal bleeding.

## Methods

A 28-year-old man was admitted to the ICU following intubation and sedation for seizures due to acute alcohol withdrawal. A CT scan of the brain was normal. In the ICU the patient became haemodynamically unstable and fresh blood was aspirated from the nasogastric tube. Gastroscopy showed bleeding oesphageal varicies and hypertensive portal gastropathy. The patient was treated with variceal banding, tazobactam/pipacillin (4.5 g three times daily) and terlipressin (2 g four times daily) as per protocol for variceal bleeding. He was successfully extubated 48 hours later.

## Results

On admission the patient's serum sodium was 139 mmol/l. The patient received terlipressin for 4 days in the following regimen: 2 g four times daily for 48 hours, then 1 mg four times daily for 24 hours and then 0.5 mg four times daily for a further 24 hours before stopping. On the last day of his terlipressin therapy, the patient's GCS dropped from 15 to 11. Serum sodium had fallen acutely to 116 mmol/l. The last two doses of terlipressin were cancelled and no other treatment for hyponatraemia was administered. His serum sodium and GCS returned to normal limits within 13 hours of terlipressin cessation with no neurological consequences.

## Conclusion

There are few case reports of terlipressin-induced hyponatraemia [1]. Hyponatraemia is considered a rare side effect brought about by the partial agonist effect of terlipressin on renal vasopressin V2 receptors. Sola and colleagues monitored serum sodium concentrations in patients with acute variceal bleeding. They found that rapid reduction in serum sodium was common (up to 36%) and one patient developed osmotic demyelination syndrome. Hyponatraemia resolved on cessation of terlipressin [2]. Close monitoring of serum sodium levels is essential in patients on terlipressin therapy so rapid drops in sodium can be identified and managed to stop associated neurological complications.
